# Description of the first sleeping sickness case diagnosed in Burkina Faso since two decades

**DOI:** 10.1371/journal.pntd.0006677

**Published:** 2018-08-20

**Authors:** Emilie Dama, Aboubacar Drabo, Jacques Kaboré, Elie Ouédraogo, Bamoro Coulibaly, Hamidou Ilboudo, Justin Kaboré, Charlie Franck Compaoré, Hassane Sakandé, Micheline Ouédraogo, Jean-Baptiste Rayaissé, Fabrice Courtin, Philippe Solano, François Drabo, Vincent Jamonneau

**Affiliations:** 1 Université Nazi Boni, Unité de Formation et de Recherche Sciences et Techniques, Bobo-Dioulasso, Burkina Faso; 2 Centre International de Recherche-Développement sur l’Elevage en zones Subhumides, Unité de recherches sur les bases biologiques de la lutte intégrée, Bobo-Dioulasso, Burkina Faso; 3 Centre Hospitalier Universitaire Yalgado Ouédraogo, Ouagadougou, Burkina Faso; 4 Programme National de Lutte contre les Maladies Tropicales Négligées, Ouagadougou, Burkina Faso; 5 Institut Pierre Richet, Unité de Recherche “Trypanosomoses”, Bouaké, Côte d’Ivoire; 6 Institut de Recherche pour le Développement, INTERTRYP, Université de Montpellier-IRD-CIRAD, Montpellier, France; Institute of Tropical Medicine, BELGIUM

## Abstract

Burkina Faso belongs to a group of countries in which human African trypanosomiasis (HAT), caused by *Trypanosoma brucei gambiense*, is no longer considered to be a public health problem. Although no native cases have been detected since 1993, there is still the risk of HAT re-emergence due to significant population movements between Burkina Faso and active HAT foci in Côte d’Ivoire. Since 2014, Burkina Faso receives support from the WHO to implement a passive surveillance program. This resulted in the detection in 2015 of the first putative native HAT case since two decades. However, epidemiological entomological and molecular biology investigations have not been able to identify with certainty the origin of this infection or to confirm that it was due to *T*. *b*. *gambiense*. This case emphasises the need to strengthen passive surveillance of the disease for sustained elimination of HAT as a public health problem in Burkina Faso.

## Author summary

In 2012, the roadmap for the Control of Neglected Tropical Diseases (NTD) of the World Health Organization (WHO) included human African trypanosomiasis (HAT) to be eliminated as a public health problem by 2020. To reach this ambitious objective in Burkina Faso, where the vector (and consequently a risk of HAT re-emergence) is still present, a passive surveillance system based on sentinel sites was established in the southwestern part of the country, considered to be the most at-risk area. The implementation of this system recently resulted in the diagnosis of the first putative native sleeping sickness case since two decades. Although the origin of this infection and how the patient was infected could not be identified, the detection of this native case confirms that HAT re-emergence in Burkina Faso is still a risk. This demonstrates the importance of implementing, maintaining and reinforcing passive surveillance programs in at-risk areas.

## Introduction

Human African trypanosomiasis (HAT), or sleeping sickness, is a neglected tropical disease caused by the protozoan parasites *Trypanosoma brucei* (*T*.*b*.) *gambiense* and *T*.*b*. *rhodesiense*, which are transmitted through the bites of infected tsetse flies (*Glossina* spp.). Control of *T*.*b*. *gambiense* HAT, assumed to be primarily a chronic anthroponosis in West and Central Africa, relies on the detection and treatment of infected cases and on vector control. As a result of intensive control campaigns and changes in land use and climate that have reduced the human reservoir of trypanosomes and tsetse populations, the prevalence of HAT has been drastically reduced in recent decades. In 2016, 2,184 HAT cases were reported to the World Health Organization (WHO), of which more than 97% were due to *T*.*b*. *gambiense* [1].

Currently in West Africa, the endemic HAT foci are localised in the coastal mangrove area of Guinea and the forest area of Côte d’Ivoire [2]. Although the savannah area of Burkina Faso was particularly affected between 1930 and 1980 with tens of thousands of cases [3], the last native case was reported in 1993 in the Bobo-Dioulasso area [4]. No cases have been reported in at-risk areas since 2005, when active mass screening was initiated [5,6]. However, a few cases imported from Côte d’Ivoire are detected every year in Burkina Faso [7]. This situation is due to the significant migratory movements of populations between these two bordering countries and their important historical, social and economic connections [8]. Thus, there is still a risk of HAT re-emergence in areas where the vector persists in Burkina Faso, mainly in the southwestern part of the country [5].

**Fig 1:**
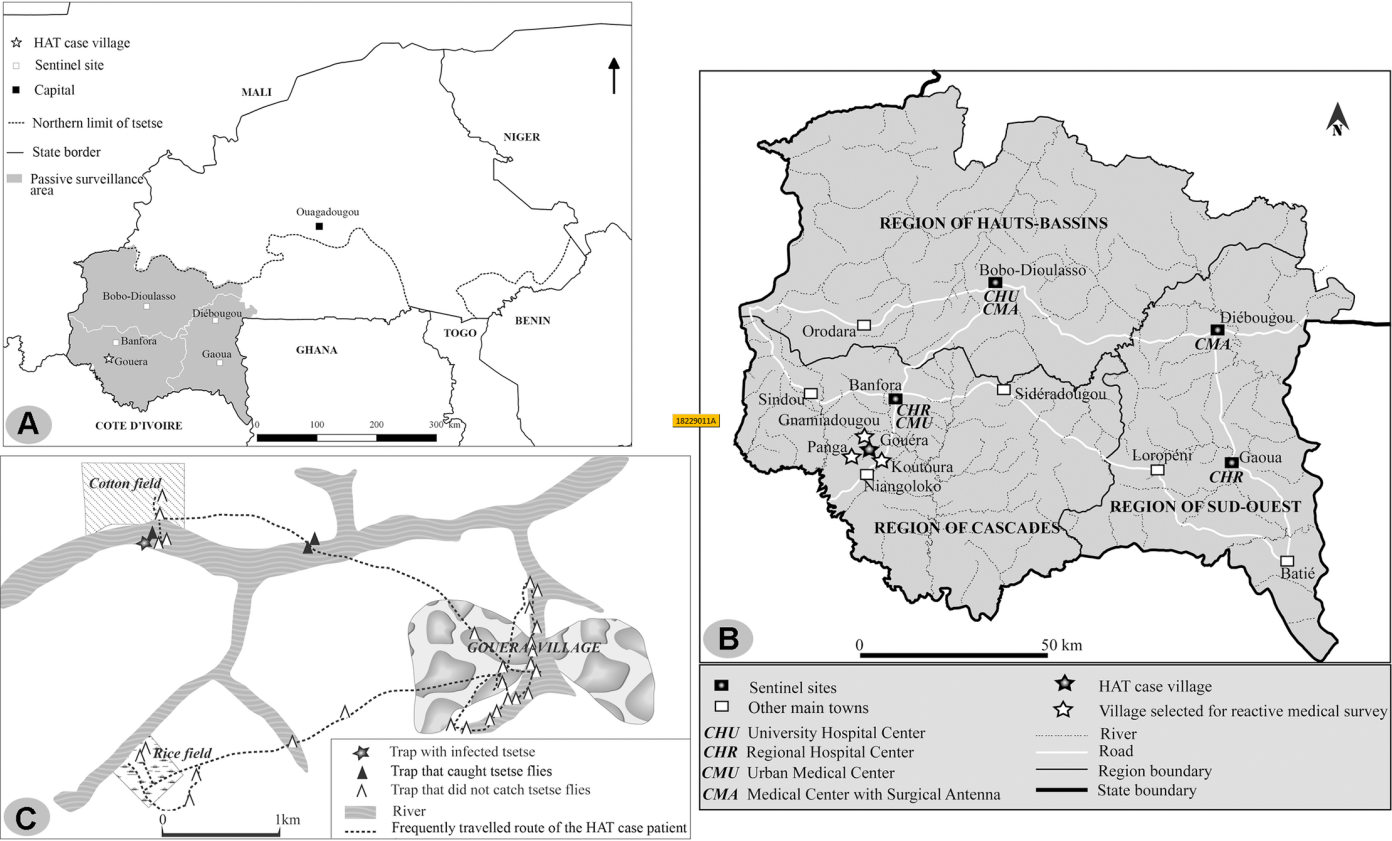
Study area and relevant epidemiological information regarding the reported case. (A) Tsetse spatial distribution and passive surveillance area in the southwestern part of Burkina Faso. (B) HAT sentinel sites and relevant geographical data. (C) Entomological survey of the daily living spaces of the patient.

In order to eliminate HAT as a public health problem by 2020 as targeted by the WHO [9], a passive surveillance system based on 6 sentinel sites was established in April 2015 in the main health referral structures of this area, which is comprised of three administrative regions (Fig 1). Two sites were selected in each of the three regions: a hospital and a peripheral health structure in Bobo-Dioulasso (Hauts-Bassin region); in Banfora (Cascades region); the hospital in Gaoua and a peripheral health structure in Diebougou (Sud-Ouest region). Health staff located at these six sentinel sites received training on the clinical suspicion of the disease as well as serological diagnosis using a Rapid Diagnostic Test (RDT). In the event of an RDT-positive subject, the blood is then sampled and spotted on filter paper (Whatman n°4) and sent to the Centre International de Recherche-Développement sur l’Elevage en zone Subhumide (CIRDES; Bobo-Dioulasso, Burkina Faso) for subsequent immune trypanolysis (TL) testing [10]. If the TL test is positive, classical parasitological assays are performed with the support of the central level and the CIRDES staff.

In August 2015, one HAT case was detected at the Banfora hospital sentinel site. Here, we describe this first probably native HAT case to be detected in Burkina Faso since 1993, as well as the investigations that were conducted to identify the contamination source and to anticipate the risk of re-emergence.

## Method

### Case description: From clinical symptoms to diagnosis and treatment

On August 10, 2015 a 14-year-old boy was admitted to the psychiatric department of the Regional Hospital of Banfora for psychiatric disorders primarily characterised by logorrhea, hypersomnolence and a persistent fever. For at least five months before admission, the patient visited several peripheral health care facilities close to his village with the same symptoms. However, no health professionals considered the possibility of HAT since the disease is no longer regarded to be a threat in this area. The symptoms then progressively became aggravated and the parents said they believed that their child was “possessed by spirits”. They visited some traditional healers but his health deteriorated further, and the parents were not sure how to save their child. Fortunately, the patient’s uncle, a teacher, convinced the parents to take him to the Regional Hospital of Banfora, a preeminent health care centre in the region.

Based on the above described signs and symptoms, and thanks to the fact that a doctor had recently been trained for HAT clinical suspicion, a HAT RDT (SD Bioline HAT) was performed on August 11, 2015 and the patient turned out to be positive. The serological suspicion was subsequently reinforced by a positive TL test (on August 17) using the LiTat 1.3 variant antigenic type (VAT) performed on blood that had been dried on filter paper and by a positive Card Agglutination Test for Trypanosomiasis (CATT; on August 19) preformed on whole blood and an end titer of 1/32 in CATT performed with twofold plasma dilutions (CATT pl). Parasitological investigations conducted on August 19 revealed the presence of trypanosomes in the blood with the mini Anion Exchange Centrifugation Technique performed on 350 μl of buffy coat [11] (20 trypanosomes) and in the cerebrospinal fluid (CSF) using the Modified Simple Centrifugation (MSC) performed with 3.5 ml of CSF [12] (5 trypanosomes). For CSF white blood cell count, a Uriglass counting chamber was used, revealing 174 cells/mm^3^. On the basis of these results, the patient was classified in stage 2 of the disease and treatment with eflornithine (DFMO), according to the national procedure, was started on August 25. The patient stayed for 2 weeks in the Regional Hospital of Banfora. His 6-month post-therapeutic follow-up confirmed that treatment was successful, as no trypanosomes were detected in the blood or CSF, fewer than 5 cells/mm^3^ were found in the CSF, and his general clinical condition was normal.

### Identification of trypanosomes

On August 19, 2015 whole blood, buffy coat (BC), plasma and CSF were collected and frozen at -20°C in the field (car freezer) and at -80°C in the lab until subsequent molecular analysis performed at CIRDES. The plasma tested positive with the immune TL test-cloned populations of *T*.*b*. *gambiense* VAT LiTat 1.3, LiTat 1.5 and LiTat 1.6, as previously described [10,13]. Briefly, 25 μl of plasma was mixed with an equal volume of guinea pig serum, to which 50 μl of a 10^7^ trypanosomes/ml suspension prepared from infected mouse blood was added. After 90 min of incubation at room temperature, the suspension was examined by microscopy (×400). The TL test was considered positive when more than 50% of the trypanosomes were lysed. TL is considered to be 100% specific for *gambiense-*HAT sero-diagnosis in humans [10].

DNA extractions from the blood, BC and CSF were performed using the QIAamp DNA Blood Mini Kit (QIAGEN). The three DNA samples tested positive with the *Trypanozoon*-specific TBR1-2 primers [14] but were negative with the *T*.*b*. *gambiense*-specific primers that target the *TgsGP* gene [15]. We unsuccessfully tried to characterise the DNA samples using four microsatellite markers (M6C8 [16], Misatg4, Misatg9 and Micbg6 [17]), probably because the method was not sensitive enough to detect and amplify small quantities of DNA. Unfortunately, the trypanosome strain could not be isolated despite inoculation of blood and CSF in six BALB/c mice that were immunosuppressed with cyclophosphamide (300 mg/kg of Endoxan^®^; administered before and then every 5 days after infection).

### Epidemiological investigations

The reported case came from the village of Gouèra, located in the Niangoloko commune in the Cascades region (Fig 1B) near the Banfora-Niangoloko road. It is close to the source of the Tanion River, which is a tributary of the Comoe River that has its source in Burkina Faso and continues its course in Côte d’Ivoire. Due to the presence of a consistent hydrological network, many transhumant cattle pass through this area on their way from Burkina Faso and Mali to the north of Côte d’Ivoire during the dry season.

Epidemiological investigations were conducted to identify the possible sources of infection. First, vertical transmission was excluded since the mother was negative for both CATT and TL. In addition, the patient never received a transfusion, thus excluding accidental infection. Family history information was then gathered, especially regarding their link with Côte d’Ivoire. From 1984 to 1994, the patient’s father, his two wives and their children (all Burkinabe people) lived in the Bonon area in Côte d’Ivoire, where they worked on coffee-cocoa plantations. Bonon is currently considered to be the most active HAT focus in the country [18]. However, the family returned to Burkina Faso in 1994 and settled in Gouera. The patient was born in this village seven years later in 2001 and never left the area since his birth, except for a 10-day stay with a traditional healer in the Ivorian village of Kawara in July 2015. Kawara is located in northern Côte d’Ivoire (near the border with Burkina Faso), far from any currently active foci. Importantly, no HAT cases have been reported in more than 20 years in this area [19]. The other members of the immediate family have not returned to Côte d’Ivoire since 1994, except for one brother who still maintains a coffee-cocoa plantation in the Man area (near the border with Guinea). No imported cases of HAT have been diagnosed in Gouera area in more than 10 years.

As with most families living in Gouera, the primary activity of the case family involves seasonal agriculture based on growing maize, millet, cotton, sesame, rice and peanut, in close proximity to the Tanion River and its tributaries. The family’s secondary activity is cattle and sheep breeding, and the patient, who does not attend school, primarily works as a herder. Although the family principally obtains water year-round from pumps and wells, the patient as well as other village herdsman (mainly young boys) often bring their animals to the rivers to graze and drink. Some people in Gouèra are also involved in agricultural activities in the coffee-cocoa plantations of the forest area of Côte d’Ivoire, mainly in the old HAT focus of Aboisso, in the southeastern part of the country.

A focus group discussion with the village authorities, elder and community-based health workers was held in Gouera village. This made it possible to observe that the population of Gouera had a limited knowledge of HAT and its transmission, although they did mention the presence of tsetse flies along the rivers near the village. However, one elder man declared himself to be a former HAT patient who received treatment in 1960.

Blood samples from 46 individuals, including all members of the patient’s family and subjects living in the same house, were tested using CATT, and three subjects were found to be positive. The three detected individuals were the first wife of the patient’s father (not the patient’s biological mother) (CATT pl = 1/2), her son (CATT pl = 1/4), and her grandson (CATT pl = 1/8). However, all three were negative for the parasitological and TL tests, thus excluding the possibility of contact with *T*.*b*. *gambiense*. In addition, microfilaria were detected in the mAECT-BC of the first wife’s son.

### Entomological investigations

The patient was monitored at his daily living spaces during both the dry and rainy seasons (Fig 1C). Specifically, the patient’s residence, the family’s fields, the water supply points, the bathing places, and the pasture points were geo-referenced and characterised. A total of 31 biconical traps were deployed for 48 hours at the most relevant sites favourable to tsetse populations. A total of 15 tsetse flies were collected in three traps, all *Glossina tachinoides*. The flies were dissected and examined by microscopy for possible trypanosome infection in the midgut, proboscis, and salivary glands. One tsetse fly was determined to be infected by trypanosomes only in the proboscis, suggesting a *Trypanosoma vivax* infection.

### Reactive medical survey

In order to identify the possible sources of HAT infection in the patient and to prevent the risk of HAT re-emergence, an exhaustive, reactive medical survey was conducted focussing on the populations of Gouera (2,781 inhabitants) and three other villages that share the same spaces (Koutoura: 2,000 inhabitants; Panga: 1,392 inhabitants; and Gnamiadougou: 2,403 inhabitants) (Fig 1B). A total of 4,214 individuals (49.13%) were tested by CATT whole blood. Out of the 27 CATT-positive subjects, 14 had a CATT plasma level ≥ 1/4 but were negative for the mini Anion Exchange Centrifugation Technique performed on buffy coat [11]. Each of the 27 CATT-positive subjects were negative for the TL test.

Simultaneously, a veterinary survey was performed and blood was collected from 46 pigs, 7 cattle and 23 sheep in the four villages. All assayed animals were negative by microscopic examination using the buffy coat technique (BCT) [20], in addition to the TL tests using the LiTat 1.3, LiTat 1.5 and LiTat 1.6 variants.

## Discussion

This case report describes a single HAT case diagnosed in 2015 in the context of a passive surveillance program implemented in the southwestern part of Burkina Faso. The area is considered to be at-risk area regarding the presence of tsetse flies, due to the significant population movements between this area and the endemic foci of Côte d’Ivoire [8]. This HAT infection case from the Banfora area is the first putative native case to be identified in more than 20 years in Burkina Faso. The medical investigations were unable to identify the source of contamination, since no other trypanosomes could be detected in humans or in a sample of domestic animals occupying the same spaces as the HAT case. These results therefore seem to exclude the re-emergence of HAT in the study area, even if we were unable to screen the total human and domestic animal populations of Gouera. However, the presence of tsetse flies was confirmed.

Concerning the identification of the trypanosome, some indicators suggest that the infection is due to *T*. *b*. *gambiense*: the clinical status of the patient, the detection of parasites in the CSF, the positive result with the *Trypanozoon*-specific TBR primers, and the positive TL result with the LiTat 1.3 and LiTat 1.5 variants. Nevertheless, this could not be confirmed by *TgsGP* PCR or by microsatellite genotyping. However, it should be noted that the corresponding primers target single copy genes and may not be sensitive enough to exclude the presence of *T*. *b*. *gambiense* in the case of a negative result obtained from biological samples, for which parasitaemia are generally low [21]. Unfortunately, the strain isolation assays failed, making it impossible to apply these primers to purified and more concentrated DNA. Regarding the well-known low virulence of *T*.*b*. *gambiense* and its low success rate of isolation in rodents [22–24], the isolation failures encountered in this study are an additional argument for characterising this as a *T*.*b*. *gambiense* infection, even if it is still not a definitive proof. To complicate matters, a recent study conducted in trypanosomes circulating in domestic animals in the HAT foci of Bonon and Sinfra in Côte d’Ivoire suggested that it cannot be excluded that *T*. *b*. *brucei* strains are capable of expressing the LiTat 1.3 variant, culminating in TL-positive results [25]. To summarise, although we strongly suspect the presence of a *T*.*b*. *gambiense* infection, we cannot exclude the possibility of a *T*.*b*. *brucei* infection associated with possible immunodeficiency. Indeed, the *T*.*b*. *brucei* parasite is normally not infectious to humans since it undergoes immediate lysis through the trypanolytic activity of the human-specific apolipoprotein L-1 (ApoL1) [26]. Nevertheless, a lack of the trypanolytic ApoL1 protein could be responsible for human infection with animal trypanosomes, as already described for one *T*. *evansi* infection in India [27] and one transient *T*.*b brucei* infection in Ghana [28]. A *T*. *evansi* infection is very unlikely in this region.

Our investigations suggest that the source of contamination may be a HAT case infected in an Ivorian focus, since part of the local population regularly stays in Côte d’Ivoire to work in the coffee-cocoa plantations, a biotope favourable to human/tsetse fly contact and where HAT still occurs [29]. However, such a case could not be detected during the medical surveys, either because no such infected individuals participated in the active case finding or because they do not live in the area anymore (*e*.*g*. they have returned to Côte d’Ivoire). The source of contamination could also be a residual local human reservoir since the area is a previous focus. It was recently observed that HAT is not invariably fatal if untreated, and that some individuals can tolerate *T*.*b*. *gambiense* without obvious symptoms for a prolonged period [30,31]; one recent case persisted for 29 years without displaying any symptoms [32]. Finally, it should not be excluded that the source of contamination may be a domestic or wild animal reservoir of *T*.*b*. *gambiense*, since the existence of such a reservoir is still under debate [25, 33]. No *T*.*b*. *gambiense* were detected in local domestic animals in this study, suggesting that this potential animal reservoir would rather be due to the transhumant cattle [34] that regularly stay in the area during the dry season or to wild animals.

The detection of this probable native case confirms the risk of HAT re-emergence in Burkina Faso and highlights the importance of maintaining a passive surveillance program in the areas at risk. More generally, it is crucial in the context of the elimination process to integrate HAT control into the national peripheral health facilities, mainly in areas of low prevalence. This case report also affirms that further studies are required to clarify the roles of population migration, latent human infections, and possible animal reservoirs of *T*.*b*. *gambiense* in the epidemiology of HAT. Studies on the movement of human and animals populations in HAT foci and the examination of animals may become part of the toolbox for post-elimination monitoring, in order to ensure sustained zero-transmission in controlled HAT foci. Finally, there is also the possibility of underestimating atypical human infections caused by trypanosomes that normally infect animals [35], and thus the ability to differentiate a *T*.*b*. *gambiense* infection from these atypical infections is also an important challenge. This illustrates the importance of developing sensitive, *T*.*b*. *gambiense*-specific tools [1].
